# Preterm Birth and Healthy Outcomes Team: the science and strategy of team-based investigation

**DOI:** 10.1186/1471-2393-13-S1-S1

**Published:** 2013-01-31

**Authors:** Suzanne C Tough

**Affiliations:** 1 Department of Paediatrics, Faculty of Medicine, University of Calgary, 2888 Shaganappi Trail NW, Calgary, Alberta, T3B 6A8, Canada; 2 Department Community Health Sciences, Faculty of Medicine, University of Calgary, 3280 Hospital Drive NW, Calgary, Alberta, T2N 4Z6, Canada

## Introduction

In the traditional academic environment, there are often more reasons not to construct a team than there are reasons to construct one. In particular, there are institutional and funder guidelines for reward and recognition that are disincentives to the creation of a team. There are temporal pressures that favor small group work and incremental science over large groups and high risk projects. In addition, we are increasingly encouraged to make our research relevant and valid while reducing the time required for the translation of evidence to practice and policy. While the need for accurate science is paramount, these requests must still be accommodated within the parameters of academic and health delivery systems in flux, reductions in research budgets, and changing government priorities.

Nevertheless, an unparalleled opportunity is created when a group of dedicated people with the desire to address a common, complex problem realize that a solution cannot be reached within the traditional paradigm. These people recognize that the solutions do not lie within the realm of a single discipline, faculty, institution, theoretical framework, or dogma, and they are willing to look for new ways of working together. Such is the complex problem of preterm birth, and such is the work of the Preterm Birth and Healthy Outcomes Team (PreHOT).

Preterm birth impacts about 9.6% of pregnancies worldwide [1,2]. Preterm birth is the major cause of neonatal death, accounting for 25-50% of deaths of infants without congenital anomalies – a total of 1 million deaths world-wide annually [1,2]. Preterm birth is also responsible for the majority of newborn morbidity including cerebral palsy, cognitive impairment, blindness, deafness, respiratory illness and complications of neonatal intensive care [3]. Together these contribute to the loss of over 100 million disability-adjusted life-years [2]. The March of Dimes estimates the cost of preterm birth in the United States to be $26 billion annually [4]. In Canada, the Canadian Institute for Health Information estimates hospital costs alone for the care of very premature babies (<28 weeks) to be more than $100 million, whereas the total long-term costs are estimated at $13.3 billion per year [5]. In addition to the basic economic costs, attending to the needs of a child with a disability requires time, care and attention from families, extended families, health care systems, schools and public services. The conscious and unconscious dreams of parents and grandparents are revisited and often revised with the delivery of a preterm baby. Preterm birth influences not just the child, but the entire family’s well being and quality of life.

## PreHOT

The impact of preterm birth reaches beyond any one discipline or area of life, and so too must the solutions. Despite decades of research, rates of preterm birth have not decreased; indeed in many regions they are increasing. Currently, there is no effective therapy to prevent preterm birth in women in true preterm labour. Clearly, a new approach to the prevention of preterm labour is urgently needed to reduce the medical, social and economic costs of preterm birth. Through the PreHOT research program, researchers have been able to take advantage of a unique, interdisciplinary approach to improve the prediction of preterm birth, to determine who will benefit from interventions, and to reduce the adverse influence of preterm birth on parents and families through interventions that optimize parenting and development for those born preterm. Ultimately, these strategies could offer hope to the millions of families annually whose lives are shattered by the death or disability of their newborn baby due to its prematurity.

To establish its direction, PreHOT investigators created a shared vision of “optimizing outcomes in preterm birth through research, intervention and knowledge translation.” Each of the investigators working on PreHOT shares a goal to increase the prediction of preterm birth, support interventions that improve outcomes for infants and parents, and finally, determine ways to improve long term outcomes for these vulnerable members of our communities. Indeed, a common vision is the driving principle of the team as we face the challenges of multidisciplinary work and timeline cooperation. Through the past five years of team development, the following have remained core values and priorities that have enabled a disparate group of scientists create an expansive and synergistic program of research together:

a) a passion for solving the complex problem of preterm birth,

b) a commitment to developing the next generation of researchers,

c) an appreciation of the time and attention required for the engagement of policy and decision makers,

d) an interest in learning from each other,

e) a willingness to be personally and professionally engaged in success,

f) a commitment to developing a business structure with working agreements and governance documents, and

g) a committed and accountable executive

The work presented in this supplement is the outcome of these five years of work and dedication from both senior and junior scientists, as well as numerous trainees. This *BMC Pregnancy and Childbirth* supplement includes samples of the research accomplished across multiple disciplines to further our understanding of the causes and consequences of preterm birth. It is our hope that the work of the team will continue over the upcoming years.

### Progress

PreHOT was formed in 2008 through funding from Alberta Innovates Health Solutions, formerly the Alberta Heritage Foundation for Medical Research, Interdisciplinary Team Grants Program (#200700595). In order to optimize functioning and outputs, extensive investment was made in developing the team ethos and integrity. These efforts were facilitated by engaging a professional coach who led team members through a consensus process to develop the vision, mission, working agreements, conflict resolution, financial management and recognition (authorship, intellectual property) plans. The engagement of team members in the creation of a team handbook was an innovative and effective approach to encourage team success. The dedication to the process enabled us to minimize communication errors and to establish team-specific operating principles and accountabilities that were acceptable across discipline, faculty, and institution. In addition, team members made meaningful time commitments to attend team meetings, get to know each other, understand the work of researchers in other content areas, engage in discussions to ensure their work was understood by others of different content expertise, and provide valuable feedback to team members regardless of discipline. Indeed, through these efforts we believe we have been able to learn from one another, increase the sophistication of our science, and identify emerging questions to be addressed across scientific paradigms, and collectively establish and maintain a high integrity, highly collaborative research environment. In addition, we have examined the learning and work styles each researcher brings to the team, which has enabled us to embrace the diversity of our unique talents, develop mutual respect, reduce frustration, and increase engagement and productivity.

Through this process of engagement with one another, PreHOT developed a governance and operational structure that encouraged communication and collaborative decision making. An executive met quarterly to review the budget and project progress and to strategize solutions for potential barriers to success (eg. delays in ethics approvals or release of funds). Research project leaders met routinely with their research teams at schedules appropriate to the project (e.g., weekly for some, monthly for others), and they provided quarterly reports to the executive members and the funder. Trainee events were scheduled twice per year and included both academic and social activities to encourage team building (eg. knowledge mobilization workshop, rafting). Team members and trainees committed to attending an annual meeting, which included administrative and progress reports as well as scientific presentations. At these annual meetings, team members participated in leadership development activities and further team building exercises.

### Knowledge mobilization

There is increasing pressure on scientists to work across disciplines and sectors to address complex problems and to increase the policy and practice relevancy of our work. To that end, our team actively sought input into the research program from stakeholders that included family advisors, health service decision makers, clinical decision makers, policy makers and government officials. Our operational model integrates knowledge end-users through the initiation of research projects to the interpretation and contextualization of our research findings. Well-established collaborations among researchers, families, and policy and decision makers ensure that our research will continue to be relevant and have great potential to improve health outcomes.

We have junior and senior decision-makers from the local health region associated with the development, implementation and interpretation of practice-relevant projects. These relationships, which promote trust and respect, ensure the relevance and impact of our findings. For example, in collaboration with the clinical community and health region, we have been able to create evidence about the effectiveness of programs and services that enhance prenatal care, support parents in caring for preterm infants in the neonatal intensive care unit, and improve father and infant interaction.

### Training and mentorship

We have provided an interdisciplinary academic environment to over 100 trainees in the past five years, allowing these trainees to gain substantive and methodological expertise to prepare them as future research experts. Trainees participate in an interdisciplinary research course where they work with those outside their discipline to create research projects and gain exposure to the broader research and policy environment through seminars and discussions. The trainees have actively evaluated this learning experience, which has been published in the peer reviewed literature [6-10]. The PreHOT operational model is strategically designed with one senior and one junior investigator on each project so that mentorship is continuous, direct and timely among the academic, clinical and policy team members. In addition, trainees are continuously provided opportunities to develop skills in leadership and knowledge mobilization. For example, trainees present their research to a cross disciplinary group which may include policy and decision makers as well as scientists from other disciplines.

### Connecting with national and international initiatives

Strong PreHOT connections have been developed with other Canadian and international research groups and networks. PreHOT provides a unique context in which to further develop preterm birth research in collaboration with national (i.e., Canadian Institutes for Health Research, Natural Sciences and Engineering Research Council of Canada) and international (i.e., WHO Preterm Birth International Consortium [PREBIC]) partners. Other collaborations focus on the opportunity to harmonize data collection or data sharing agreements with other researchers and research teams to facilitate longitudinal comparisons, increase methodological efficiencies, and answer our research questions with increased accuracy and timeliness.

### Accomplishments

Since coming together five years ago, PreHOT members have produced over 250 publications (190 of which were peer-reviewed), over 400 presentations and 160 outreach activities with academic and stakeholder audiences. All these outputs are related to predicting, preventing and improving the well-being of children and families affected by preterm birth.

PreHOT’s network of collaborations stretch far beyond the core team through numerous research collaborations, policy related committee and panel memberships, and dedicated trainees. Members of the team have initiated projects with the Alberta Epigenetics Network to learn more about how stress could program our genetic expression. The All Our Babies Study has connected with the RAINE Study in Western Australia, the National Children’s Study, Centering Healthcare Institute, the Alberta Centre for Child Family and Community Research and Alberta Health Services in order to share and enhance understanding about maternal and child health. The guinea pig model of premature labour developed within the team resulted from collaboration with the University of Newcastle (UK) and University of Texas Southwestern Medical Center through their investigations of the uterine mechanisms related to preterm birth. Investigators from PreHOT contribute their academic expertise to policy related committees and panels at the local (UpStart, Royal Alexandra Hospital Research Advisory Committee), provincial (Alberta Health Services - Population Health Working Group, Women’s Reproductive/Preconception Health Committee and the newly released Child Health Strategic Clinical Network, Preconception Health Committee, and the Centre for Child Well-Being Advisory Committee), national (Canadian Public Health Association, Maternal-Infant and Youth Research Network, Canadian Centre for Behavioral Neuroscience, Canadian Neonatal Follow-up Network, Assisted Human Reproduction Canada Scientific Advisory Panel, Canadian Collaboration for Prenatal Health Research, and the Child and Youth Mental Health Initiative), and international (International Preterm Birth Collaborative and the Worldwide Universities Network) levels. Our graduate students and postdoctoral fellows have been very successful in applying their training to further academic pursuits and scientific positions with institutions across the continent including Alberta Health Services, University of Calgary, McMaster University, and Wayne State University.

## Research progress

Over the past five years, PreHOT research has centered around the mechanisms, pathways, and health service delivery related to preterm birth. This research program is designed as a complementary series of synergistic investigations, alternating between hypothesis testing in animal and human models. The research represents significant advancement toward achieving the overarching vision of the team—healthy pregnancies and birth outcomes in a healthy community—by advancing the specific mission goals of creating leading edge multidisciplinary evidence to:

a) reduce the incidence and consequences of preterm birth,

b) inform effective interventions that reduce preterm birth, and

c) support clinical, community and policy use to mitigate the risk and impact of preterm birth.

In addition, the research demonstrates the team’s commitment to training and capacity building through the many trainee contributions, as well as knowledge mobilization. This supplement includes research findings that illustrates the breadth of the work and highlights the influence interdisciplinary teamwork can have on solving complex problems.

With a balance across the pillars of health research, PreHOT addresses research questions in three thematic areas: (a) prediction of preterm birth, (b) prevention of preterm birth, and (c) interventions related to preterm birth, which are each represented through articles in this supplement. (a) Within the first thematic area, members of our team have been working on a number of projects, three of which are represented in this supplement. In order to learn more about predicting preterm birth, we have established the All Our Babies Longitudinal Cohort to better investigate early life influences on preterm birth, child development and family well-being [11]. This cohort can assist in discovering the relative influence of the social environment compared to the genetic environment on the risk of preterm birth. In addition, the prospective longitudinal nature of the cohort will permit identification of the earliest markers of child and family risk so that interventions are implemented closer to the root cause for improved success. Thus far, the cohort has also allowed us to explore the nature and reliability of self-reported data in contrast to medical records [12], and to develop tools to better identify women at risk of poor mental health (previously published) [13]. Concluding this section, a related topic in the prediction of preterm birth is represented in a paper on the implications of the genome wide association studies on preterm birth for obstetricians-gynecologists [14]. (b) To address the second theme on prevention of preterm birth, the team has a diversity of projects from animal models to group prenatal care models. Two papers address the group prenatal care model called Centering Pregnancy in terms of the impact of the program in comparison to traditional prenatal care and education [15] and the experience of physicians providing the program [16]. Investigations of uterine cells (myometrium) will enable understanding of the interactions between maternal and fetal physiological systems in human term and preterm labour [17]. Another study examines how maternal circulating leukocytes display early chemotactic responsiveness during late gestation [18]. Additional examinations of the molecular physiology of spontaneous preterm birth in rat and guinea pig models are aligned with human longitudinal studies. With samples from the longitudinal rat study, we are also examining the predictive biomarkers for preterm birth and undertaking controlled laboratory experiments to study the intergenerational influence of preterm birth [19]. (c) Within the final theme on interventions related to preterm birth, we are exploring the experiences of preterm birth and testing the effectiveness of related, innovative interventions with the potential for economic benefits. A systematic review completed by team members is the first to attempt to untangle the effects of intervention components on parent and preterm child outcomes over the short and longer term [20]. Additional work on this theme is examining depressive symptoms among mothers with high risk preterm infants [21]. Specific interventions include studies of parent-provided care in the neonatal nursery [22], and a program to improve father and preterm infant interactions. A final article also presents an interpretive description of the experiences of parents with preterm infants [23].

## Summary

The solutions to complex health and social problems compel us to collaborate interdisciplinary approaches and to encourage cross-sector thinking. The current time required to translate science into practice can be abbreviated through partnership and engagement. The opportunity for high quality science to be used as evidence in decision making is contingent on researchers seeking opportunities to be invited to the decision making table. PreHOT, with support from its partners and funders, has attempted to create a research platform that can respond to emerging scientific discoveries as well as clinical and program needs related to preterm birth. Through a team approach and a shared vision, PreHOT has been inspired to develop new methods and processes to address the complex issue of preterm birth with the hope of reducing the burden of adverse birth outcomes on families and society.

## Competing interests

The authors declare that they have no competing interests.

## Declarations

This article has been published as part of *BMC Pregnancy and Childbirth* Volume 13 Supplement 1, 2013: Preterm Birth: Interdisciplinary Research from the Preterm Birth and Healthy Outcomes Team (PreHOT). The full contents of the supplement are available online at http://www.biomedcentral.com/bmcpregnancychildbirth/supplements/13/S1.

All of the publication fees will be funded by the Preterm Birth and Healthy Outcomes Team Interdisciplinary Team Grant (#200700595) from Alberta Innovates - Health Solutions, formerly the Alberta Heritage Foundation for Medical Research.
